# Multisensory integration of musical emotion perception in singing

**DOI:** 10.1007/s00426-021-01637-9

**Published:** 2022-01-10

**Authors:** Elke B. Lange, Jens Fünderich, Hartmut Grimm

**Affiliations:** 1 Department of Music, Max Planck Institute for Empirical Aesthetics (MPIEA), Grüneburgweg 14, 60322 Frankfurt/M., Germany; 2 Present Address: University of Erfurt, Erfurt, Germany

## Abstract

We investigated how visual and auditory information contributes to emotion communication during singing. Classically trained singers applied two different facial expressions (expressive/suppressed) to pieces from their song and opera repertoire. Recordings of the singers were evaluated by laypersons or experts, presented to them in three different modes: auditory, visual, and audio–visual. A manipulation check confirmed that the singers succeeded in manipulating the face while keeping the sound highly expressive. Analyses focused on whether the visual difference or the auditory concordance between the two versions determined perception of the audio–visual stimuli. When evaluating expressive intensity or emotional content a clear effect of visual dominance showed. Experts made more use of the visual cues than laypersons. Consistency measures between uni-modal and multimodal presentations did not explain the visual dominance. The evaluation of seriousness was applied as a control. The uni-modal stimuli were rated as expected, but multisensory evaluations converged without visual dominance. Our study demonstrates that long-term knowledge and task context affect multisensory integration. Even though singers’ orofacial movements are dominated by sound production, their facial expressions can communicate emotions composed into the music, and observes do not rely on audio information instead. Studies such as ours are important to understand multisensory integration in applied settings.

**Supplementary Information:**

The online version contains supplementary material available at 10.1007/s00426-021-01637-9.

## Introduction

The perception of emotional expressions serves a central function in human communication and has behavioral consequences, e.g., associated emotional and physical responses (Blair, 2003; Buck, 1994; Darwin, 1872; Ekman, 1993). With emotion expression and communication at its core, music is a substantial part of everyday life (Juslin & Laukka, 2004; North et al., 2004), serving cognitive, emotional, and social functions (Hargreaves & North, 1999). As musical performance is often perceived in conjunction with visual input (e.g., concerts, music videos), understanding how emotion is communicated involves understanding the specific impact of different sensory modalities. However, multisensory perception of emotion is understudied (Schreuder et al., 2016). We investigate emotion communication in singing performance as one applied setting of multisensory emotion perception.

In music performance, multisensory emotion communication has mainly been investigated with regard to two aspects: the general impact of the visual modality, and the specific effect of an expressive musical interpretation. *Visual dominance* has been reported for musical performance quality (Tsay, 2013), expressivity (Vuoskoski et al., 2014), perceived tension (Vines et al., 2006), perceived emotional intensity (Vuoskoski et al., 2016) or valence (Livingstone et al., 2015), and for different instrumentalists’ and singing performances (Coutinho & Scherer, 2017; Livingstone et al., 2015). The majority of studies addresses the strong impact of visual cues, but auditory cues can be of importance when the visual information is less reliable (i.e., point-light animations, Vuoskoski et al., 2014, 2016), or when combining music with emotional pictures or unrelated films (Baumgartner et al., 2006; Marin et al., 2012; Van den Stock et al., 2009).

The second aspect relates to the perception of the expressivity of musicians’ performance (e.g., Broughton & Stevens, 2009; Davidson, 1993; Vines et al., 2011; Vuoskoski et al., 2016). In these studies, expressivity was manipulated in a holistic way without specifying facial expression. The exact patterns of results did not replicate between the studies, but some general patterns emerged. Low expressivity affected evaluations of musical performance rather more than an exaggerated one, and effects appeared more clearly in audio–visual stimuli than in auditory-only stimuli, indicating that visual cues can further strengthen the expression composed into the music.

Our study investigates how emotional content and intensity is communicated during singing and extends earlier ones in several respects. First, our focus is on facial expression as one key feature in emotion communication (Buck, 1994; Ekman, 1993). Note that some studies on musical emotion communication filtered the face to remove facial expression as potentially confounding information (e.g., Broughton & Stevens, 2009; Dahl & Friberg, 2007), but systematic research on an impact of such facial information is underrepresented.

Second, we decided on singers because singing requires to open the mouth widely, which has the emotional connotation of anger, fear, or surprise (Darwin, 1872; Ekman, 1993). In addition, sound-producing and ancillary orofacial movements are particularly interlinked in singing (Livingstone et al., 2015; Siegwart-Zesiger & Scherer, 1995). This might limit singers’ options to express emotions by their faces and cause problems for the observers to decode the emotions expressed in the musical performance. As a result, auditory information might become more important for audio–visual perception of singing performance.

Third, we assume that musical performance is a situation in which blended emotions, rather than discrete basic emotions, are expressed and perceived (Cowen et al., 2020; Larsen & Stastny, 2011). In music, basic emotions can co-occur (Hunter et al., 2008; Larsen & Stastny, 2011), and are difficult to be differentiated (e.g., fear in Dahl & Friberg, 2007; sadness and tenderness in Gabrielsson & Juslin, 1996). Some research takes the wide variety of musical expression into account (Coutinho & Scherer, 2017; Juslin & Laukka, 2004; Zentner et al., 2008), but often studies on musical expression use a small range of categories (Gabrielsson & Juslin, 1996; Juslin & Laukka, 2003). To capture the blended and rich emotional experiences in music perception, we based our selection of emotion expressions on a hermeneutic analysis of the music by musicologists.

Fourth, since emotion expression serves an important function, and since music communicates emotions (Gabrielsson & Juslin, 1996; Juslin & Laukka, 2003, 2004), one might expect not only experts but also laypersons to be able to decode emotion expressions in music (Bigand et al., 2005). But musical training changes how music or sound is perceived (e.g., Besson et al., 2007; Neuhaus et al., 2006), and how emotional cues and expressivity are extracted from music or tone sequences (Battcock & Schutz, 2021; Bhatara et al., 2011; Broughton & Stevens, 2009; Thompson et al., 2004). It is thus important to clarify the role of expertise for emotion communication in music. Hence, we included a layperson-expert comparison.

In general, singers’ facial expressions are still underrepresented in studies of emotion communication, but the number of studies is growing. Qualitative case studies of music videos (Thompson et al., 2005) point to performer-specific functions of facial expressions, such as displaying affect or regulating the performance-audience interaction. Quantitative studies investigated the effect of singers’ facial expressions on pitch perception (Thompson et al., 2010), on the emotional connotations of sung ascending major or minor thirds (Thompson et al., 2008) or of a single sung vowel (Scotto di Carlo & Guaïtella, 2004). Livingstone et al. (2009) showed a clear difference for happy or sad facial expressions when singing a seven-tone sequence. Decoding accuracies for a limited set of emotion expressions were high for speech and only slightly worse (Livingstone et al., 2015) or similar for song (Livingstone & Russo, 2018). This indicates that the sound-producing orofacial movements of singing might not be devastating for emotion communication. Coutinho and Scherer (2017) investigated felt emotions of singing performance in a concert setting, extending research to real music and using a 28-item questionnaire with 12 classes of emotions and no focus on facial expressions. The similarity of the induced emotion profile for visual-only stimuli and the audio–visual stimuli were rather high, and higher than the similarity between the auditory-only and audio–visual stimuli. This indicates a strong contribution of visual information to the emotional experiences of the crossmodal stimuli.

## Rationale of the study design

**Fig. 1:**
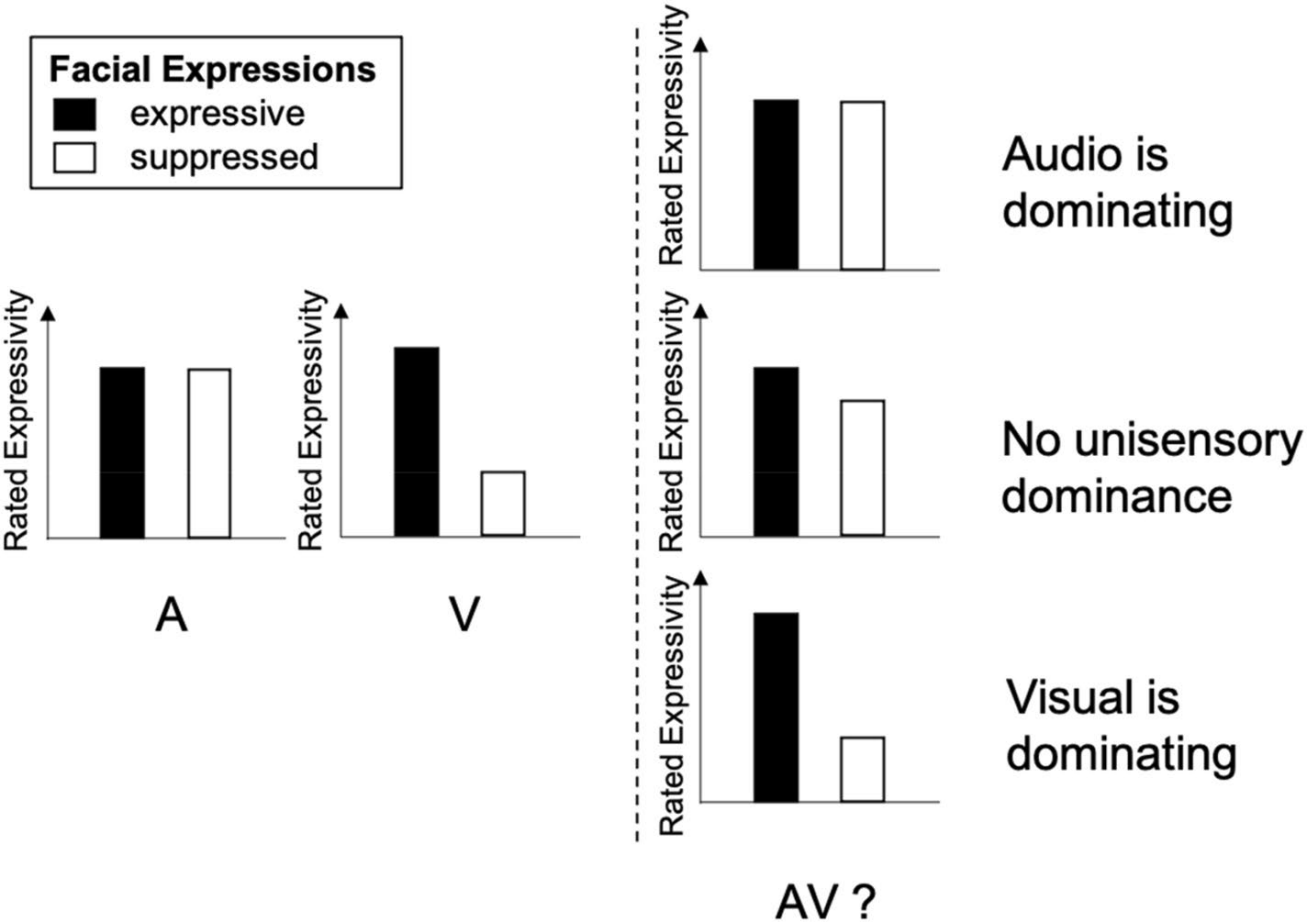
Graphical depiction of the logical account that tested for *visual dominance*. On the left of the dashed line (A and V), predicted results for the manipulation check. On the right (AV?), three hypothetical outcomes for the evaluation of audio–visual performance (AV). Facial expressions were expressive (solid black) or suppressed (solid white)

We presented recordings of singers and asked participants to evaluate perceived intensity and content of communicated emotions in three presentation modes: visual, auditory, or audio–visual. Unlike other studies (Broughton & Stevens, 2009; Davidson, 1993; Vines et al., 2011), we specifically instructed the singers to manipulate their facial expressions, singing with either expressive or suppressed facial expressions, while keeping the musical interpretation the same. Highly skilled singers from one of the leading German conservatories of music were trained by a professor of acting and the recordings were controlled by a professor of music aesthetics and a video artist. We expected ratings of intensity and emotion expressions for the visual stimuli to be higher in the expressive condition than in the suppressed one, but to remain the same for the auditory stimuli. Upon this manipulation check of the singers’ instructions, the critical tests were on how information from the two uni-sensory modalities were combined into an integrated percept of the auditory–visual stimuli (Fig. 1). The rationale is that perception of the combined stimuli is the direct consequence of the evaluations of its visual and auditory components. If the visual stream dominated, the strong effect of facial expression would transfer to the audio–visual stimuli (*visual dominance*). If the auditory stream dominated the audio–visual stimuli, there would be a small or no effect of facial expression in the evaluation of the audio–visual stimuli (*auditory dominance*). Finally, information from the senses could be merged without uni-sensory dominance.

Given the demonstrated *visual dominance* for performing instrumental musicians (e.g., Tsay, 2013), we expected *visual dominance* to show in our setting as well. However, given that intended emotional facial expressions during singing might be contaminated by the singer’s sound-producing orofacial movements, listeners might rely on auditory cues (*auditory dominance*) or do not weight one sense over the other (e.g., see Van den Stock et al., 2009; Vuoskoski et al., 2014). That is, if we succeed in showing *visual dominance*, listeners were able to decode and make-use of emotional facial expressions despite the fact that sound-producing orofacial movements might interfere with the emotional facial expression.

To test our predictions, we applied a series of interactions of ANOVAs, including facial expression as one factor and a subset of the three presentation modes as second factor. Including the factor facial expression (expressive, suppressed) and the two uni-sensory presentation modes (A, V) should result in a significant two-way interaction, confirming the successful manipulation. More importantly, we predicted a significant two-way interaction between the factor facial expression and the auditory and audio–visual presentation modes (A, AV) and at the same time no significant interaction between the factor facial expression and the visual and audio–visual presentation modes (V, AV). This exact pattern of two interactions indicated *visual dominance* of the facial expression (see the section “Method, Statistical tests” for more information on the series of statistical tests). To further evaluate sensory dominance, we analyzed the consistency of ratings between the uni-sensory and crossmodal stimuli (Coutinho & Scherer, 2017).

In accordance with other studies, we assumed music experts to rely on visual and not auditory cues (e.g., Tsay, 2013). To understand whether experts make even more use of visual cues than laypersons, we prepared audio–visual stimuli that were re-combined from the original two recordings, one with expressive the other with suppressed facial expression. If experts make more use of visual cues in audio–visual perception, exchange of the visual part should interact with expertise.

Note that one interesting manipulation would be to change expressivity in singing, e.g., suppressing emotion expression in the audio but keeping the face expressive. However, there are practical reasons that preclude such manipulation. Whereas the human face can arguably be “emotionless” and facial expressions “neutral”, complex music (e.g., from opera) cannot be without emotional expression (e.g., see Akkermans et al., 2019, their Fig. 1 and Table 5, showing that an “expressionless” interpretation was similarly rated as sad, tender, solemn, and the acoustic features expressing “expressionless” overlapped highly with happy interpretations). There is no valid manipulation of music precluding emotional expressivity. There is also no possibility to make the manipulation of emotional expressivity in sound comparable to the manipulation of the visual.

## Method

### Participants

Participants received a small honorarium of 10 Euro per hour. They gave written informed consent and performed in two sessions, each about 60 min long. The experimental procedures were ethically approved by the Ethics Council of the Max Planck Society.

An a priori statistical power analysis was performed for sample size estimation for the within-group comparisons with GPower (Faul et al., 2007). With an assumed medium effect size (*f *= 0.25, *f*′ = 0.3536), *α* = 0.05, power = 0.80), and a low correlation of *r* = 0.3 the interaction of the two factors presentation mode (with two levels from A, V, AV) and facial expression (expressive, suppressed) would need at least 24 participants. Between-group comparisons require *n* ≥ 30 per group (central limit theorem). We planned 32 participants for each group, based on the balancing scheme of conditions. Final sample size for the laypersons deviated slightly due to practical issues in recruiting (overbooking). Post hoc power evaluations (Lakens & Caldwell, 2019; http://shiny.ieis.tue.nl/anova_power/) showed high power to detect the two-way interactions within groups (all power calculations > 90%).

#### Laypersons

Thirty-four students from Goethe University, Frankfurt am Main, Germany, were recruited (seven male, mean age *M* = 23, diverse fields of study with four students from psychology, and no from music or musicology). The mean *General Musical Sophistication* (Müllensiefen et al., 2014) of the sample was *M* = 77.67 (SD = 15.70). Participants were not enthusiasts of opera or lieder/song recitals, as was established by eight questions on musical taste, listening habits and the frequency of concert visits.

### Experts

Thirty-two students were recruited at music academies and musicology departments in Frankfurt and surrounding cities (ten male, mean age *M* = 26). Twenty-one of them were studying music (Bachelor and Master); ten were pursuing the Master in Musicology; and one responded with “other”—but we decided to be conservative and keep the data of this person in the set. The mean *General Musical Sophistication* was *M* = 93.60 (SD = 13.02), which was significantly higher than for the laypersons, *t*(63) = 4.44, *p* < 0.001, *η*^2^ = 0.239. Note that the distributions of the feature “musical sophistication” overlapped between groups and we defined here experts by their profession. The experts reported stronger liking of listening to song recitals and more visits to the opera and song recitals than did the laypersons. They also watched videos of operas or song recitals more often, all *t*s > 2.9, *p*s < 0.05, α Bonferroni corrected).

### Apparatus

Data collection took place in a group testing room, with a maximum of four participants tested in parallel, each seated in a separate cubicle equipped with a Windows PC, monitor, and Beyerdynamic headphones (DT 770 Pro 80 Ohm). Stimulus presentation and data collection were programmed in PsychoPy 1.82.01 (Peirce, 2007). Loudness was set to the same comfortable level for all participants.

### Materials: stimuli

#### Video recordings

**Fig. 2:**
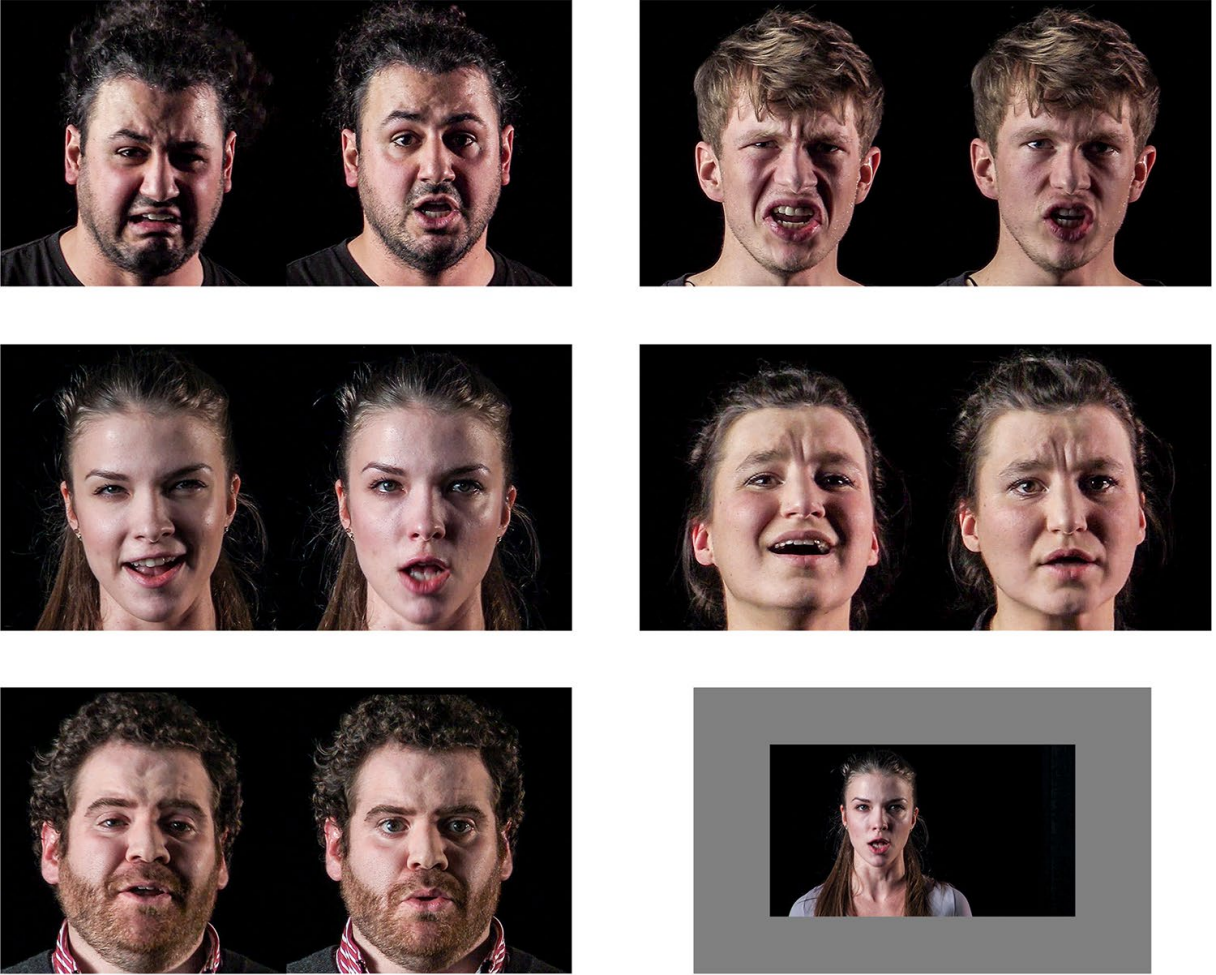
Examples of expressive (left) and suppressed (right) facial expression for all five singers. The images shown here are stills from the stimulus video recordings used in the study, with the aspect ratio slightly modified to emphasize the faces. The double-image stills were from the exact same moment in the music sang in the two different versions. The lower right subplot is an example for the original size of the video on the monitor

Five singers from the *Hochschule für Musik Hanns Eisler Berlin*, Germany, were asked to fulfill two tasks: (1) sing with expressive face and gestures, and (2) sing with suppressed facial or gestural expression, “without anything” (Fig. 2).^1^ Each singer performed two, self-chosen musical pieces (composers: Händel, Schumann, Offenbach, Puccini, Mahler, de Falla, Strauss, and Britten) in both conditions, accompanied by a professional repetiteur. The recording session with each singer was completed when the recording team (consisting of a professor for acting, a professor for music aesthetics [author H.G.], and a video artist) were satisfied with the two versions.

The videos were recorded with two Canon XA25 cameras (28 Mb/s; AVCHD format; 50 frames per second). Singers were recorded frontally, close-up (head and shoulders), with a distance of about 9.5 m between them and the cameras. A professional lighting technician set up lighting equipment to focus the recordings on the singer’s facial expressions, while the background appeared infinite black. The sound recordings of the singers were taken by two microphones (Neumann KM 185) in x–y-stereophony.

#### Selection of excerpts

From the basic material of the recordings, we (authors J.F., H.G., and E.L.) selected 15 excerpts based on the following criteria and a consensus decision-making process: (a) as little change in musical expression as possible within the musical excerpt, (b) high facial expressivity in the expressive condition, (c) synchrony between the visual and auditory streams when swapped between videos (i.e., visual part from the recording in the suppressive condition and auditory part from the expressive condition or vice versa), (d) overall quality of the performance, and (e) keeping at least two excerpts per singer in the set (see Supplementary Material, Table S2, for a list of all excerpts).

#### Stimulus types created from the recordings

We then created eight stimuli from each chosen excerpt. We coded stimuli from the expressive face condition with 1, and from the suppressed condition with 0, the uni-modal stimuli with one letter (A or V) and the audio–visual with two letters (AV), resulting in a: A1, b: A0, c: V1, d: V0, e: A1V1, f: A0V0, and the swapped stimuli g: A1V0 and h: A0V1. Note that in the suppressed condition the visuals were without expression, but the auditory part was as expressive as in the other condition (by instruction), that is A0 codes expressive audio, recorded with suppressed facial expression. The eight stimuli (a–h), extracted from the 15 passages, resulted in a total of 120 excerpts. Mean length of excerpts was about 15 s (range: 9–23 s). Note that predictions in Fig. 1 refer to the stimuli a–f. The two swapped videos (g, h) were included to test for a stronger impact of visual cues for experts than laypersons.

### Materials: questionnaire on musical expressivity

We assembled a 12-item questionnaire on musical expressivity. Eleven items captured emotional expressions and were based on a traditional, hermeneutic musicological analysis, explained below. Ten terms were chosen for the expressive stimuli: *anger*,* cheekiness*, *disappointment*,* tenderness*,* pain*,* longing*,* joy*,* contempt*,* desperation*, and *sadness*; one term was selected as relevant for suppressed facial expression: *seriousness*. In addition, we included the *intensity of expressivity* (“Ausdrucksintensität”). We also included *ineffability/indeterminacy* (“Unbestimmtheit/Das Unbestimmbare”), a term widely discussed in music aesthetics (Jankélévitch, 1961/2003). But participants had difficulties to understand this term. Due to its low validity, we had to exclude this term from analyses.

All items were rated on seven-point Likert-scales. For the content items, scales measured whether a specific expression was communicated by the performer, from 1 (*not at all/rarely*) to 7 (*very*). The intensity scale measured the overall intensity of expressivity irrespective of the content.

To assemble the content items, we followed a multi-step procedure. First, we asked two professional musicologists to analyze the complete musical pieces and generate verbal labels describing the composed emotional content based on the score and the audio files. Second, we gathered all verbal expressions, assembled clusters of semantic content in fields, converted the material into nouns, resulting in 30 terms: *seriousness*,* melancholy*, *emphasis*, *power*, *pain*, *sadness*, *longing*, *entreaty*, *insecurity*, *aplomb*, *tenderness*, *dreaminess*, *desperation*, *tentativeness*, *horror*, *resignation*, *timorousness*, *anger*, *disappointment*, *agitation*, *reverie*, *hope*, *cheekiness*, *cheerfulness*, *lightheartedness*, *relaxation*, *contempt*, *being-in-love*, *joy*, and *indeterminacy*. Third, we visited a musicology class at Goethe University with 13 students attending. We presented a random selection of 21 original excerpts (A1V1 or A0V0). Each student monitored the videos for seven or eight of the 30 terms and responded binomially, yes or no, as to whether these terms were relevant semantic categories for describing the perceived musical expressions. From these results, we assembled the final, eleven content items with the additional goal of keeping the list diverse. Fourth, one student, trained in psychology and music, piloted the 120 stimuli on the eleven items. This person knew the full list of 30 terms and was asked to identify any important terms that might be missing from the final item selection, which was not the case.

Note that the selection procedure was mainly based on expert knowledge. Culture- and style-specific knowledge plays an important role to recognize musical emotions (Laukka et al., 2013). That is, comparing evaluations between experts and laypersons will particularly show how laypersons will differ from such expert coding.

### Procedure

Data were collected in two sessions. The session started with the assessment of the *General Musical Sophistication* and the questionnaire on listening habits regarding opera and song recitals (see Participant section), split and counterbalanced across sessions. The evaluations of the excerpts followed, with 60 trials in each session. All participants evaluated all 120 stimuli. The start of each trial was self-paced, and self-chosen breaks were allowed at any time. The videos were presented centrally on a PC monitor with a gray background screen (covering about 60% of the screen, 1280 × 720 pixel, see lower right image in Fig. 2). When presenting the auditory-only conditions, a black placeholder of the same size as the video was shown on the monitor. After presentation, each stimulus was evaluated by the questionnaire, with all items visible on the PC display at the same time.

Excerpts were presented in blocks of ten trials, keeping the presentation mode (A, V, AV) the same within a given block. Within blocks, the different conditions were mixed (i.e., expressive or suppressed, original or swapped). The selection of excerpts for each block was randomized for participants, with one random selection matching between one participant of the layperson group and one of the experts group. The sequence of the blocks was balanced using a complex Latin square design to reduce serial order and serial position effects of the presentation mode (completely balanced for *n* = multiples of 8 participants).

### Data treatment

#### Emotion expression: composite score

One simple way to calculate how strongly emotions were communicated would be to average across the ten content items per trial (excluding *seriousness* as key expression for the suppressed condition and the *intensity* measure). However, emotions were often regarded to not be expressed (i.e., mode of one; see Supplementary Materials, Figure S1), indicating that the emotional content of each piece was captured by a selection of items with high inter-individual differences. Averaging across these ratings weights the high number of low ratings, thus shifting the mean towards a lower value, overestimating ratings for which the expression was not present and reducing possible differences between conditions. We, therefore, defined the most relevant expressions per piece post hoc from the collected data on the A1V1 stimuli, and averaged across this selection (see Supplementary Materials, Figure S2).

We based relevance on two definitions: (1) individual relevance: the participant’s rating of four or higher (four is the midpoint of the 1–7 scale), and (2) general relevance: at least 1/3 of all participants evaluated the item as relevant (rating of four and higher).^2^ For the composite score, we averaged across the relevant items, considering the ratings of all participants for the entire range of 1–7. Some stimuli communicated a small range of four blended emotions up to a blend of all ten item (see Supplementary Materials, Table S3, for the relevant emotional expressions of each stimulus). The blends were a composite of very heterogenous emotions (e.g., *anger*, *cheekiness*, *longing*, and *joy* for stimulus 5).

#### Statistical tests

Ratings were treated as continuous variables and analyzed by three-factorial, mixed-design analyses of variance (ANOVA), using the statistical package SPSS, version 26. The two within-subject factors were presentation mode and facial expression, the between-subject factor was expertise. *α* was set to 0.05, testing was two-sided. We tested the logical account outlined in Fig. 1 by a series of hypothesized, a priori, two-way interactions, each including the manipulation of facial expression as one factor and a selection of the three presentation modes as the other (see Table 1).

**Table 1:** Statistical hypotheses testing

	(1) Presentation mode (A, V) × facial expression (1, 0)	(2a) Presentation mode (A, AV) × facial expression (1, 0)	(2b) Presentation mode (V, AV) × facial expression (1, 0)
Manipulation check			
Stronger effect of the facial expression manipulation in V than A	Significant	–	–
Sensory dominance			
Audio is dominating	Significant	Not significant	Significant
Visual is dominating	Significant	Significant	Not significant
No dominance	Significant	Significant	Significant

Presentation modes with one letter decode uni-sensory presentation, with two audio–visual presentation. Recordings were with expressive (1) or suppressed (0) facial expression. Stimuli a– f (see “Method”) included in this sequential testing account

The successful manipulation was tested by the two-way interaction between the facial expression (expressive or suppressed) and presentation mode including the uni-sensory stimuli (A, V; Table 1: column 1). Changing the facial expression should affect the perceived expressivity of visual recordings but not of the auditory recordings, corresponding to the instructions given to the singers. Upon successful manipulation, we tested for *visual dominance*: Two two-way interactions were evaluated at the same time (Table 1: columns 2a and 2b). For *auditory dominance* there would be an interaction between facial expression and presentation mode in (2b) but not in (2a), and for *visual dominance* an interaction in (2a) but not in (2b). If there was no uni-sensory dominance, but instead a fusion of the senses without dominance, both interactions would be significant. By implementing expertise as third factor in the ANOVAs, we tested whether the critical two-way interactions were modulated by the experimental group, which would be demonstrated by three-way interactions.

To further study multisensory integration, we calculated the consistency between different modes of presentation. For *visual dominance*, consistency between evaluations of the visual and the auditory–visual stimuli were assumed to be high, and for *auditory dominance* between the auditory and the auditory–visual stimuli. Consistency refers to a relative agreement (higher or lower evaluations) but not absolute agreement. We applied an intraclass correlation coefficient (ICC) measure to calculate inter-mode consistency.^3^ Instead consistency between *k* raters on *n* objects, we computed consistency between each two modes of presentation on 165 single ratings (15 stimuli × 11 content scales). We computed these consistencies for each participant separately and report the mean across participants. We calculated ICCs using one-way random effect models (e.g., type ICC(1,1), Shrout & Fleiss, 1979), using the *irr* package in *R* (Gamer et al., 2019; R Core Team, 2019).

Finally, we conducted an analysis of the original and re-combined audio–visual stimuli. Here, we make comparisons within one presentation mode only, the audio–visual mode. The successful manipulation of singers’ instructions will be mirrored by comparing stimuli for which the auditory part remained the same, but the visual was exchanged (from recordings of the expressed or suppressed condition), e.g., comparing A1V1 and A1V0. For these comparisons, perceived expressivity should be higher when the visual part came from the expressive videos. When keeping the visual part the same and exchange the auditory (e.g., comparing A1V1 and A0V1), evaluations should be comparable. If experts made better use of visual information in audio–visual stimuli, the visual manipulation should have a stronger effect for experts than laypersons. Accordingly, we predicted a two-way interaction of the factor visual manipulation and expertise in the three-factor ANOVA (auditory manipulation, visual manipulation, expertise).

## Results

### Visual dominance

A complete list of results of the three three-factor ANOVAs are reported in the Appendix, Tables 3, 4 and 5. To reduce complexity, we report here results on the interactions that test our a priori hypotheses only (Fig. 1, Table 1).

#### Intensity ratings

**Fig. 3:**
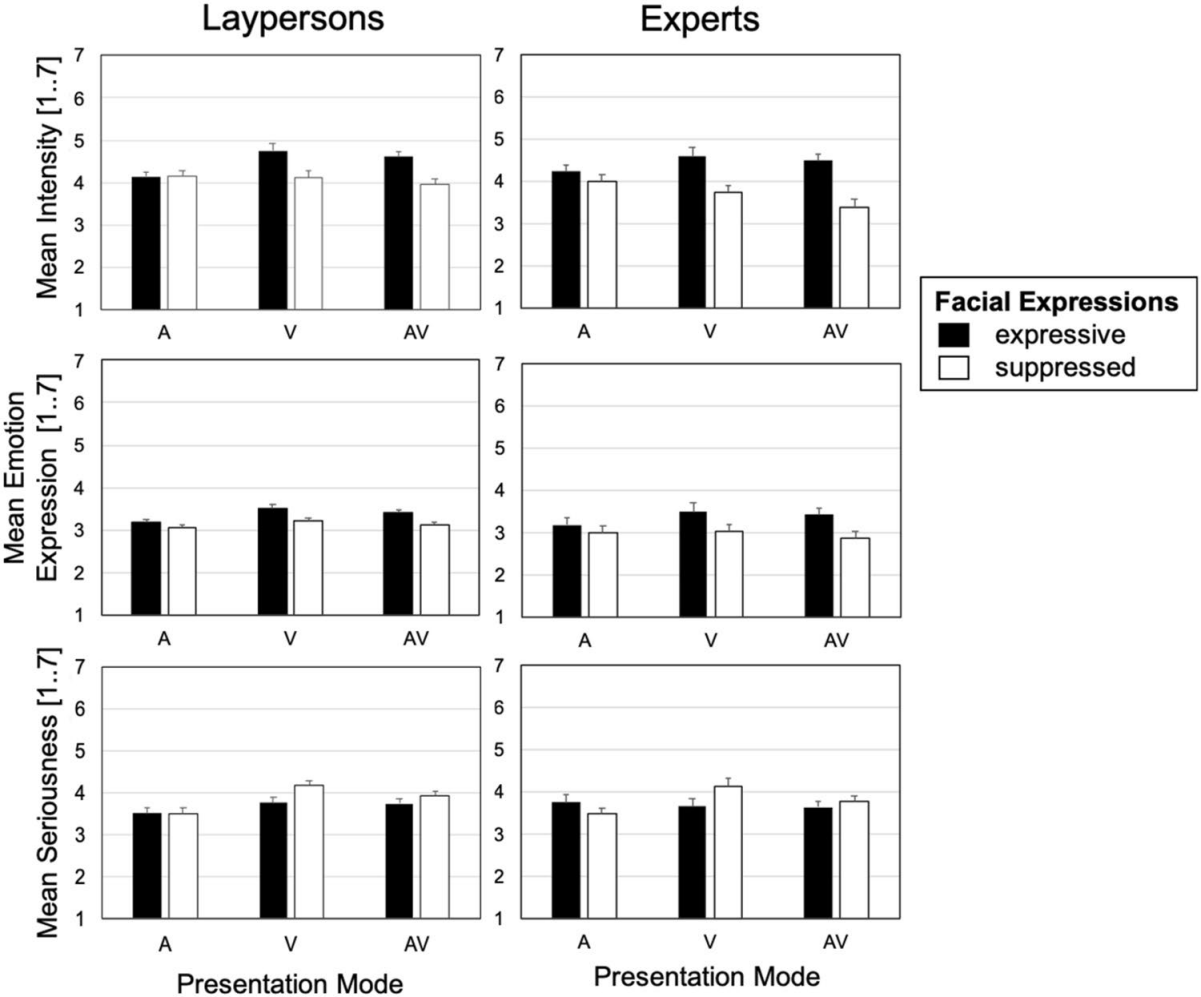
Communicated intensity, emotion expressions, and seriousness for the auditory (A), visual (V), and audio–visual (original) stimuli (AV). The upper panels show results on mean intensity, the middle on mean emotion expression (the composite score), the lower on mean seriousness, measured by a 7-point scale, with results for laypeople and experts presented in columns, stimuli types a–f. Error bars depict 95% confidence intervals adjusted for between-subject variability for within-subject comparisons (Bakeman & McArthur, 1996), separately for laypersons and experts

Results of the three critical interactions confirm what Fig. 3, upper panel, depicts. Singers succeeded in their task instructions*.* Changing facial expression affected ratings for the visual stimuli but not (or to a less extend) in the auditory, *F*(1, 64) = 36.56, *p* < 0.001, *η*^2^ = 0.364. Given this result for the uni-sensory stimuli, the next two interactions are informative about multisensory perception. Results are clear and demonstrate *visual dominance*. The interaction comparing the effect of facial expression for the modes A and AV was significant, *F*(1, 64) = 57.96, *p* < 0.001, *η*^2^ = 0.475, but the other interaction not (comparing the effect of facial expression for V and AV), *F*(1, 64) = 1.24, *p* = 0.270, *η*^2^ = 0.019.

#### Emotion expression

Taking the emotion expression composite score as the dependent variable showed a very similar picture (Fig. 3, middle panel). The manipulation was successful, the interaction with presentation modes A and V was significant, *F*(1, 64) = 23.76, *p* < 0.001, *η*^2^ = 0.271. Next, we tested for specific sensory dominance. The interaction with presentation modes A and AV was significant, *F*(1, 64) = 55.17, *p* < 0.001, *η*^2^ = 0.463, the other with presentation modes V and AV not, *F*(1, 64) = 1.60, *p* = 0.211, *η*^2^ = 0.024. That is, we replicated *visual dominance.*

Similar results for emotional content and intensity might be not surprising, as even the emotional content variable included an intensity aspect (e.g., a specific emotion being more or less communicated). However, the replication is important to note as the result of *visual dominance* replicated between two very different measures (intensity based on one item, the emotion composite core based on 4–10 items) and subject groups (laypersons, experts).

#### Seriousness

The variable seriousness served as a control. We expected the ratings of seriousness to show an opposite effect of facial expression, that is increased communication of seriousness in the suppressed condition. Indeed, this is what Fig. 3, lower panel, displays and what the ANOVA with uni-modal presentation modes confirmed. The factors presentation mode (A, V) and facial expression interacted significantly, *F*(1, 64) = 29.04, *p* < 0.001, *η*^2^ = 0.312. However, Fig. 3, lower panel, indicates that for seriousness there is no clear pattern of *visual dominance*. Both critical interactions were significant, *F*(1, 64) = 8.03, *p* = 0.006, *η*^2^ = 0.112 for presentation modes A and AV, and *F*(1, 64) = 10.02, *p* = 0.002, *η*^2^ = 0.135 for V and AV. The audio–visual percept was not dominated by a single sensory stream but integrated without dominance.

#### Facial expression

It is noteworthy to mention that there is a tendency for the two-way interactions between the factor facial expression and expertise to be significant for the ANOVAs with intensity or emotion expression as dependent variable, but not for seriousness (Appendix Tables 3, 4 and 5). Together with Fig. 3 from the main text, this indicates that the manipulation of facial expressions affected experts’ evaluations on intensity and emotion expression more than laypersons. However, the exact pattern of these results is difficult to interpret, as conditions overlapped between ANOVAs. However, to test for the hypothesis that experts made more use of the visual cues, we will compare the evaluations of the original and swapped stimuli.

### Consistency of evaluations between presentation modes

We asked whether *visual dominance* is related to the consistency with which emotional content was perceived in the stimuli. Then, consistency between evaluations of the visual-only and the auditory–visual stimuli should be higher in comparison to lower consistency between evaluations for auditory-only and auditory–visual stimuli. We calculated the ICCs for all eleven emotional-content ratings on all stimuli between each two presentation modes for each participant (see “Method”). Table 2 reports the consistency for the four comparisons as mean ICCs across participants. Consistency was overall low. The complexity of the setting (composed songs or opera music, a broad range of emotional items to capture the blended emotion account) likely contributed to such low consistency. However, the confidence intervals show that consistency was well above zero (no consistency), so there is some systematic evaluation of the stimuli. We fitted the consistency data into a three-factor mixed ANOVA with the within-subject factors of between-mode comparison (consistency between A and AV, consistency between V and AV) and facial expression (expressive, suppressed), and experimental group as between-subject factor. The factor facial expression was significant, *F*(1, 64) = 9.19, *p* = 0.004, *η*^2^ = 0.126, but mode comparisons and experimental group not, both *F*s < 1. No interaction was significant, all *F*s ≤ 3.92, *p*s ≥ .052. That is, consistency was overall lower for comparisons including suppressed facial expressions (No. 1 and 3 in Table 2) than for comparisons including expressive faces (No. 2 and 4 in Table 2). But consistency between the visual-only and crossmodal stimuli was not higher than consistency between the auditory-only and crossmodal stimuli. That is, even though the patterns of ANOVAs on expressivity and emotion expression reported above demonstrated *visual dominance*, this dominance was not related to the consistency of evaluations. It rather seems that the evaluation criteria differed for recordings presented in different modes (A, V, AV).

**Table 2:** Consistency of evaluations between different presentation modes

No.	Consistency between two presentation modes	Laypersons	Experts
1	V0 and V0A0	0.286 (0.228–0.344)	0.265 (0.197–0.333)
2	V1 and V1A1	0.346 (0.290–0.403)	0.313 (0.245–0.382)
3	A0 and V0A0	0.314 (0.239–0.390)	0.305 (0.242–0.368)
4	A1 and V1A1	0.300 (0.230–0.371)	0.341 (0.268–0.414)

Means across participants, with 95% confidence intervals in brackets. Stimuli recorded with suppressed facial expressions are coded as 0 and with expressive faces as 1

### Visual cues and the role of expertise

The original and swapped videos were fitted into mixed ANOVAs with the within-subject factors auditory component (A taking from the AV stimuli, recorded either with suppressed or expressive facial expressions), visual component (V taking from the AV stimuli, both facial conditions), and the between-subject factor expertise. We report the critical main effect of the visual component and its interaction with expertise for all three dependent variables here and a complete list of results in the Appendix (Table 6).

**Fig. 4:**
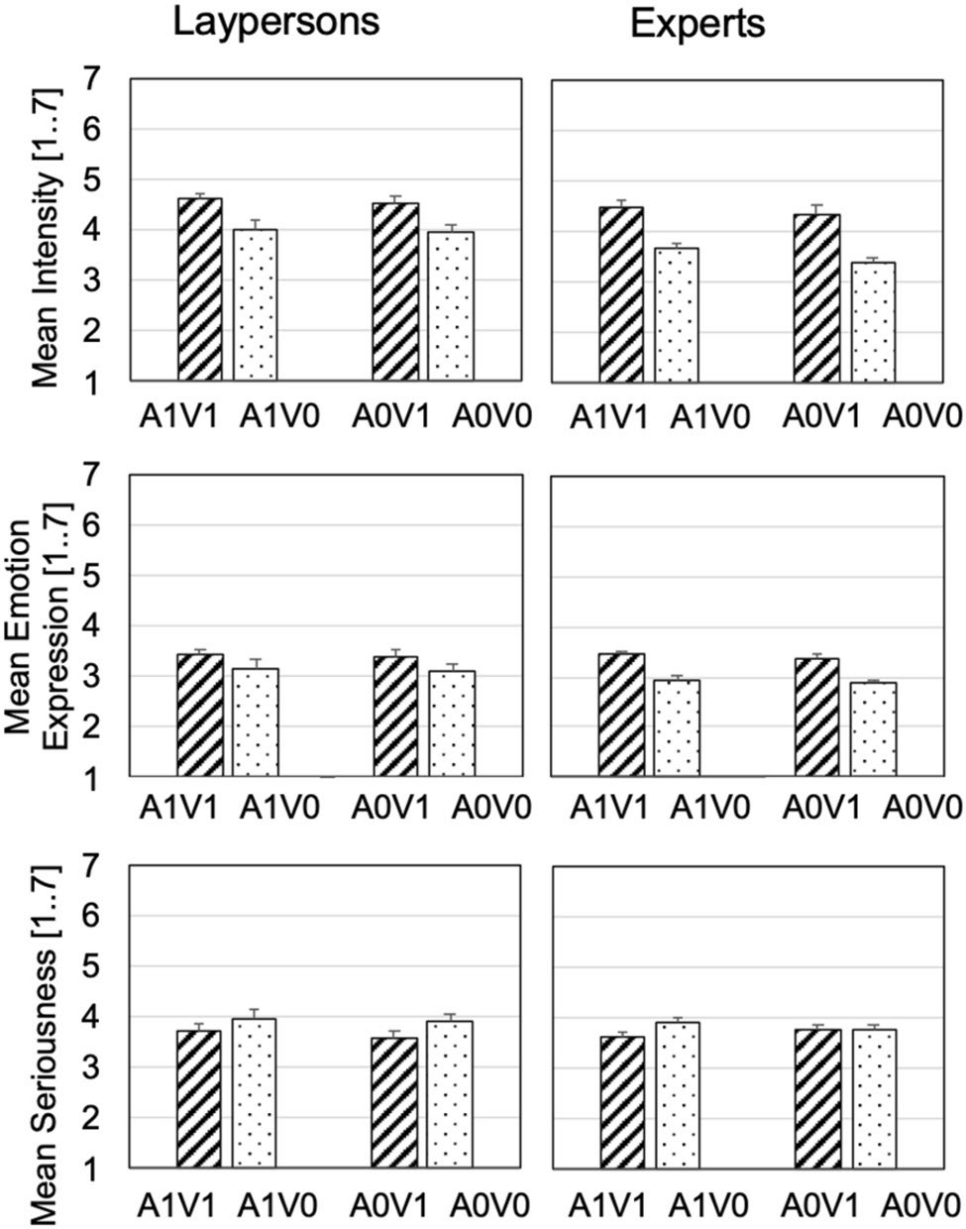
Communicated intensity, emotion expressions and seriousness for the original or swapped audio–visual stimuli (AV). The upper panels show results on mean intensity, the middle on mean emotion expression (the composite score), the lower on mean seriousness, measured by a 7-point scale, with results for laypeople and experts presented in columns, stimuli types e–h; striped pattern for the visual recording from the expressive version and dotted pattern for visual recordings from the suppressed version. Error bars depict 95% confidence intervals adjusted for between-subject variability for within-subject comparisons (Bakeman & McArthur, 1996), separately for laypersons and experts

For the perceived expressive intensity, exchanging the visual component was significant, *F*(1, 64) = 91.08, *p* < 0.001, *η*^2^ = 0.587, demonstrating an effect of (visual) facial expression, in accordance to the manipulation check reported earlier. There was a tendency for this factor to interact with expertise, *F*(1, 64) = 3.68, *p* = 0.060, *η*^2^ = 0.054, and no three-way interaction, *F* < 1 (Fig. 4, upper panel). For emotion expressions, again, exchanging the visual component was significant, *F*(1, 64) = 104.38, *p* < 0.001, *η*^2^ = 0.620, and interacted with expertise, *F*(1, 64) = 9.23, *p* = 0.003, *η*^2^ = 0.126. That is, here, expertise clearly modulated the effect of facial expression, indicating that visual cues affected experts’ evaluations of the audio–visual stimuli more than laypersons (Fig. 4, middle panel). The three-way interaction was not significant, *F* < 1. For the variable seriousness, the pattern deviated (Fig. 4, lower panel). Exchange of the visual component was significant, *F*(1, 64) = 12.42, *p* = 0.001, *η*^2^ = 0.163, but it clearly did not interact with expertise, *F*(1, 64) = 1.26, *p* = 0.266, *η*^2^ = 0.019. However, the three-way interaction was significant, *F*(1, 64) = 4.34, *p* = 0.041, *η*^2^ = 0.163. Together with Fig. 4, lower panel, this indicates a peculiarity for the experts: when exchanging the visual component but keeping the auditory component from the recordings with suppressed facial expression, the evaluation of seriousness did not change (see right-most columns in Fig. 4, lower panel). Hence, in this case, experts’ evaluations of seriousness were unaffected by the visual manipulation.

## Discussion

We studied auditory–visual interactions in emotion communication. Multisensory perception of emotion communication is understudied (e.g., Schreuder et al., 2016). We used complex stimuli (music) in an applied setting (singing performance) to gain knowledge of auditory–visual interactions in more real-life experiences, a request that has been posed in the past (de Gelder & Bertelson, 2003; Spence, 2007). Other sudies on music performance have shown that visual information seems to dominate visual-auditory perception (e.g., Tsay, 2013). But perception of communicated emotions might differ for singing performance. In human interaction, facial expressions are important cues for emotion communication (Buck, 1994). During singing, sound-producing orofacial movements might interfere with proper decoding of emotional facial expressions. We tested for *visual dominance* of perceived emotional expressivity of singing performance, and the beneficial use of visual cues by experts.

Professional opera singers were instructed to sing expressively, either with expressive faces or suppressed facial expressions. The recorded performance was evaluated by laypersons and experts for different presentation modes: auditory, visual, auditory–visual. The pattern of a series of tested interactions demonstrated *visual dominance* for perceived intensity and emotional expressions in the audio–visual stimuli. When presenting original and swapped videos, experts showed a stronger effect of the manipulated facial expression than laypersons, indicating that experts made stronger use of the visual cues. Results for seriousness showed a different pattern. There was multisensory integration without visual or auditory dominance and for a specific condition, when the audio–visual stimuli contained audio recordings from the suppressed condition, experts were not affected by visual cues.

In summary, *visual dominance* is not hardwired in emotion perception of musical performance, but depends on the type of evaluation, task context, and individual differences of the audience in musical training. Importantly, we showed *visual dominance*, even though emotional facial expression is contaminated by sound-producing orofacial movements in singing performance. This indicates that observers have learned in their past to handle this difficulty, otherwise they should have relied more on the audio and not the visuals. It is unclear, why observers showed *visual dominance*. Ratings were not more consistent between the visual and audio–visual stimuli in comparison to the auditory and audio–visual stimuli. Hence, we found no evidence for a higher reliability of visual information. Rather, musical performances that combine visuals and audio result in slightly different musical representations than those from audio-only or visual-only information. The importance of multisensory and other context information to create listeners’ musical representations has also been discussed in the recent musicological literature: Music is more than the sound. In particular, music transfers meaning “by weaving many different kinds of representations together” (Bohlman, 2005, p.216), which in our case refers to visuals in addition to the audio.

Another observation indicates that our participants were able to differentiate between the intended emotional expression and sound-producing orofacial movements. Open the mouth widely is associated with negative emotions such as anger (Ekman, 1993). Angry cues from the face attract attention (Kret et al., 2013). We analysed the most relevant emotional expression for the composite score of emotion expression (Table S3, Supplementary Materials). However, not all but a large percentage (12 of 15) stimuli expressed anger. Instead, longing was included in the composite for all stimuli. It has been demonstrated that facial emotion processing is fairly early (70–140 ms after stimulus onset), but can be modulated by task instructions that direct attention to different features of the stimuli (Ho et al., 2015). We cannot rule out cuing of negative emotions by the visual processing of singing activity (or utility of negative cuing for singers in a specific musical style, e.g., metal and rock). But in our study observers either did not attend to the angry cues or filtered them out for at least three of the stimuli, that is, the task context does play an important role in multisensory integration (e.g., knowing that singing includes a widely open mouth). This conclusion is in line with accounts pointing to the importance of context for decoding facial expression (Aviezer et al., 2017), and speaks against hardwired, automated recognition of facial expressions (Tcherkassof & Dupré, 2020).

It seems counterintuitive that experts for music show *visual dominance* in the evaluation of musical expressivity and even a stronger effect than the laypersons. Music is first of all an auditory stimulus. But our findings converge with studies showing that experts are prone to visual effects (Tsay, 2013). For emotional intensity and content, experts made more use of visual cues than laypersons. This corresponds to findings that musical expertise shapes perception of musical gesture-sound combinations (Petrini et al., 2009; Wöllner & Cañal-Bruland, 2010).

An interesting extension of our research would be to move to real concert settings (see Coutinho & Scherer, 2017; Scherer et al., 2019). Singers’ faces will be less relevant, because many listeners are seated far from the stage, and body movements (Davidson, 2012; Thompson et al., 2005) might be of more importance for the evaluation of musical expressivity. In addition, our stimuli were original compositions that included sung text in different languages (German, English, Italian), and hence contained additional cues of text semantics and speech sounds to understand the emotional content of the vocal music.We did not manipulate these features (e.g., sung lyrics versus hummed, known versus unknown language), and it would be interesting to investigate the role of lyrics in addition to the expression of musical sound and faces. In the absence of lyrics (e.g., hummed song) listeners might even rely more on decoding the facial expression, resulting in a stronger effect of *visual dominance*.

Which theories account for *visual dominance* and how does our study relate to them? First, the modality-appropriateness hypothesis (Welch & Warren, 1980) states that perception is directed to the modality that provides more accurate information regarding a feature (Ernst & Banks, 2002). Our finding of *visual dominance* is compatible with the appropriateness hypothesis. It has been demonstrated that facial expressions are particularly suitable to communicate emotions, i.e., via a direct pathway and without higher cognitive retrieval processes (de Gelder et al., 2000, 2002; Pourtois et al., 2005). However, it would make little sense to argue that musical emotions are best communicated by visual cues alone or that the visual modality is more appropriate for communicating musical emotions, rendering music irrelevant for musical emotion communication. Rather, emotions expressed in music can be shaped and specified by the visual input of the performer’s facial expressions. This argument is supported by findings showing that seeing body movements can enhance the communication of musical structure (Vines et al., 2006).

Another account, one particularly attuned to *visual dominance* effects, proposes that attention can easily be captured by auditory stimuli and is predominantly directed to vision due to capacity limitations (Posner et al., 1976). However, it is not clear how this can be related to our study. We asked participants to evaluate the crossmodal stimuli holistically. Other studies asked participants to focus attention on one sense or another, showing automatic processing of the unattended stream when facial expression had to be categorized in a non-musical task (e.g., Collignon et al., 2008; de Gelder & Vroomen, 2000). A strong automatized processing, however, was not found for musical stimuli (Vuoskoski et al., 2014).

Finally, we turn to effects of congruency. In general, congruency between information from different senses benefits multisensory integration and the percept of unity (Chen & Spence, 2017). More specifically, face-voice congruency benefits emotion communication (Collignon et al., 2008; Massaro & Egan, 1996; Pan et al., 2019; Van den Stock et al., 2009). In our audio–visual material, there are two ways to understand congruency. We can compare ratings for the original videos (A1V1, A0V0) with the swapped videos (A1V0, A0V1). Congruency would then refer to the fact the sound and image was recorded at the same time. These comparisons do not result in a systematic increase of expressivity for originals (Fig. 4). But singers were ask to sing expressively even when they suppressed facial expressions. Then, the auditory–visual stimuli can be interpreted as congruent, when the highly expressive singing was accompanied by expressive facial expressions (A1V1, A0V1), and as incongruent, when singing was combined with suppressed facial expressions (A1V0, A0V0), and ratings were for intensity and emotion expressions. Indeed, results shown in Fig. 4, upper and middle panel, are compatible with the hypotheses that congruency played a role. However, for seriousness, the audio–visual materials cannot be split into congruent or incongruent conditions, and the differences in the evaluations cannot be explained (Fig. 4, lower panel).

In summary, vision has a strong impact on the communication of emotional expressions in song. We replicated the *visual dominance* effect in a complex setting and showed the importance of singers’ facial expressions, for laypersons and even more so for experts. The applications of our findings are far-reaching, spanning from the education of opera singers at music academies to the production of music videos. There is a growing tendency to listen to music via audio–visual media, such as music videos or live-streaming opera. Expressivity and emotion communication are of high relevance for the individual in selecting music and affect aesthetic judgments (Juslin, 2013). In this respect, to be seen performing appears to be highly relevant for a singer’s success. It is, therefore, important for singers to be aware of these issues and to be able to employ facial expressions in a controlled way to communicate emotional expressions effectively.

### Electronic supplementary material

Below is the link to the electronic supplementary material.

Supplementary file1 (PDF 237 KB)

## Data Availability

The data that support the findings of this study are openly available at https://osf.io/nf9g4/.

## Appendix

This is a full report of the three-way ANOVAs. In the main text only those interactions were reported, that tested the a priori hypotheses (Tables 3, 4, 5 and 6).

**Table 3:** Results of the three three-factor ANOVAs for intensity

	*F*(1, 64)	*p*	*η* ^2^
ANOVA 1: manipulation check			
Presentation mode (A, V)	4.71	0.034*	0.069
Presentation mode × expertise	2.30	0.134	0.035
Facial expression	68.98	< 0.001*	0.519
Facial expression × expertise	4.46	0.039*	0.065
** Presentation mode** × **facial expression**	36.56	< 0.001*	0.364
Pres. mode × facial expression × expertise	0.02	0.877	0.000
Expertise	0.62	0.433	0.010
ANOVA 2a			
Presentation mode (A, AV)	0.03	0.860	0
Presentation mode × expertise	6.87	0.011*	0.097
Facial expression	91.22	< 0.001*	0.588
Facial expression × expertise	11.25	0.001*	0.150
** Presentation mode** × **facial expression**	57.96	< 0.001*	0.475
Pres. mode × facial expression × expertise	1.17	0.283	0.018
Expertise	0.97	0.328	0.015
ANOVA 2b			
Presentation mode (V, AV)	8.44	0.005*	0.117
Presentation mode × expertise	0.35	0.555	0.005
Facial expression	134.72	< .001*	0.678
Facial expression × expertise	5.52	0.022*	0.079
** Presentation mode** × **facial expression**	1.24	0.270	0.019
Presentation mode × facial expression × expertise	1.54	0.219	0.024
Expertise	2.20	0.143	0.033

The critical two-way interactions are marked by bold font

*Marks significant results with *p* < 0.05 in all tables

**Table 4:** Results of the three three-factor ANOVAs for emotion expression (composite score)

	*F*(1, 64)	*p*	*η* ^2^
ANOVA 1: manipulation check			
Presentation mode (A, V)	33.89	< 0.001*	0.346
Presentation mode × expertise	0.89	0.350	0.014
Facial expression	92.37	< 0.001*	0.591
Facial expression × expertise	3.83	0.06	0.056
** Presentation mode** × **facial expression**	23.76	< 0.001*	0.271
Presentation mode × facial expression × expertise	0.85	0.360	0.013
Expertise	0.12	0.729	0.002
ANOVA 2a			
Presentation mode (A, AV)	14.99	< 0.001*	0.190
Presentation mode × expertise	2.82	0.098	0.042
Facial expression	118.48	< 0.001*	0.649
Facial expression × expertise	9.21	0.003*	0.126
** Presentation mode** × **facial expression**	55.17	< 0.001*	0.463
Presentation mode × facial expression × expertise	6.63	0.012*	0.094
Expertise	0.18	0.675	0.003
ANOVA 2b			
Presentation mode (V, AV)	9.93	0.002*	0.134
Presentation mode × expertise	0.13	0.722	0.002
Facial expression	151.58	< 0.001*	0.703
Facial expression × expertise	9.74	0.003*	0.132
** Presentation mode** × **facial expression**	1.60	0.211	0.024
Presentation × facial expression × expertise	1.62	0.207	0.025
Expertise	0.37	0.543	0.006

The critical two-way interactions are marked by bold font

*Marks significant results with *p* < 0.05

**Table 5:** Results of the three three-factor ANOVAs for the seriousness

	*F*(1, 64)	*p*	*η* ^2^
ANOVA 1: manipulation check			
Presentation mode (A, V)	41.50	< 0.001*	0.393
Presentation mode × expertise	2.41	0.126	0.036
Facial expression	6.28	0.015*	0.089
Facial expression × expertise	0.654	0.422	0.010
** Presentation mode** × **facial expression**	29.04	< 0.001*	0.312
Presentation mode × facial expression × expertise	2.06	0.156	0.031
Expertise	0.01	0.916	0.000
ANOVA 2a			
Presentation mode (A, AV)	14.13	< 0.001*	0.181
Presentation mode × expertise	4.68	0.034*	0.068
Facial expression	0.002	0.968	0.000
Facial expression × expertise	2.22	0.141	0.034
** Presentation mode** × **facial expression**	8.03	0.006*	0.112
Presentation × facial expression × expertise	0.937	0.337	0.014
Expertise	0.000	0.995	0.000
ANOVA 2a			
Presentation mode (V, AV)	13.31	0.001*	0.172
Presentation mode × expertise	0.35	0.554	0.006
Facial expression	16.84	< 0.001*	0.208
Facial expression × expertise	0.000	0.997	0.000
** Presentation mode** × **facial expression**	10.02	0.002*	0.135
Presentation mode × facial expression × expertise	0.38	0.539	0.006
Expertise	0.105	0.747	0.002

The critical two-way interactions are marked by bold font

*Marks significant results with *p* < 0.05

**Table 6:** Results of the three three-factor ANOVAs including evaluations on original and swapped audio–visual stimuli

	*F*(1, 64)	*p*	*η* ^2^
Expressivity			
Auditory component	5.91	0.018*	0.085
Auditory component × expertise	2.35	0.130	0.035
Visual component	91.08	< 0.001*	0.587
** Visual component** × **expertise**	3.68	0.060	0.054
Auditory × visual component	0.14	0.707	0.002
Auditory × visual component × expertise	0.718	0.400	0.011
Expertise	2.17	0.146	0.033
Emotion expressions			
Auditory component	9.51	0.003*	0.129
Auditory component × expertise	1.00	0.322	0.015
Visual component	104.38	< 0.001*	0.620
** Visual component** × **expertise**	9.23	0.003*	0.126
Auditory × visual component	0.01	0.927	0.000
Auditory × visual component × expertise	0.09	0.771	0.001
Expertise	0.424	0.517	0.007
Seriousness			
Auditory component	1.31	0.257	0.020
Auditory component × expertise	0.49	0.485	0.008
Visual component	12.42	0.001*	0.163
** Visual component** × **expertise**	1.26	2.66	0.019
Auditory × visual component	0.876	0.353	0.013
Auditory × visual component × expertise	4.34	0.041*	0.063
Expertise	0.01	0.925	0.000

The critical two-way interactions are marked by bold font

*Marks significant results with *p* <0.05

## Footnotes

1 We originally aimed to differentiate between the impact of facial expressions and hand gestures, but had to leave out this factor due to only limited changes in gestural interpretations between conditions. Although we interpreted the data in terms of facial expressions, upper body and head movements were not controlled and may have contributed to the expressive interpretation.

2 We also checked for a stricter criterion of relevance, i.e., for more than one half of all participants with ratings of four and higher. Indeed, such a strong criterion increased the difference on *emotion expression* between the suppressed and expressive condition, but we decided to use the weaker criterion to not overdetermine our results.

3 We decided on the ICC measurement, because we were interested in consistency of the ratings not total agreement, for which several other measures would have been more common, e.g., Cohen’s Kappa, Krippendorff’s alpha. We report in the Supplementary Materials further inter-rater agreement measures.
