# Supplementary material for: Multisensory integration of musical emotion perception in singing
Source: Psychol Res. 2022 Jan 10;86(7):2099–114. doi: 10.1007/s00426-021-01637-9 (PMC9470688; doi:10.1007/s00426-021-01637-9)
Supplement: Supplementary file 1 — Supplementary file1 (PDF 237 KB) [file 426_2021_1637_MOESM1_ESM.pdf]

## Supplementary Materials

We include here more detailed information and additional analyses for the interested reader. We will begin with more information on the Material, followed by reports of additional analyses. We report the three-way ANOVAs, including all three presentation modes depicted in Figure 3 in the main text. We added more analyses on the reliability of the ratings.

**Content of the Supplementary Materials**

**Material Section**

I) Table S1: A complete list of eight stimulus types

II) Original German terms for the questionnaire on musical expressivity

III) Table S2: A complete list of the 15 musical experts (composer, musical piece, singer)

IV) Table S3: Most relevant emotional expressions for each stimulus (1–15). Those were averaged to build the composite score of emotion expression.

V) Figure S1: Histograms for the ratings of crossmodal stimuli in the expressive face condition for (A) laypersons and (B) experts.

VI) Figure S2: Histograms for the composite score for laypersons and experts.

**Result Section**

VII) Report of the three-way ANOVAs taking all three presentation mode into account at the same time, Tables S4, S5, S6

VIII) More calculations of the reliability of evaluations (ICC, inter-rater agreement, Shrout & Fleiss, 1979), Tables S7, S8, S9

# Materials

## I) Table S1

*Complete List of the Eight Stimulus Types*

| | Abbr. | Presentation mode | Sensory information | Combined (if applicable) | Facial expression during recording |
|---|---|---|---|---|---|
| (a) | A1 | Auditory | Uni-sensory | - | Expressive |
| (b) | A0 | Auditory | Uni-sensory | - | Suppressed |
| (c) | V1 | Visual | Uni-sensory | - | Expressive |
| (d) | V0 | Visual | Uni-sensory | - | Suppressed |
| (e) | A1V1 | Audio-visual | Combined | Original | Expressive |
| (f) | A0V0 | Audio-visual | Combined | Original | Suppressed |
| (g) | A1V0 | Audio-visual | Combined | Swapped | Audio from expressive condition, video from suppressed condition |
| (h) | A0V1 | Audio-visual | Combined | Swapped | Audio from suppressed condition, video from expressive condition |

## II) Original German Terms for the Questionnaire on Musical Expressivity

The eleven items in the questionnaire on emotional expressions and were based on a traditional, hermeneutic musicological analysis. Ten terms were chosen for the expressive stimuli: *anger* (German: “Wut”), *cheekiness* (“Keckheit”), *disappointment* (“Enttäuschung”), *tenderness* (“Zärtlichkeit”), *pain* (“Schmerz”), *longing* (“Sehnsucht”), *joy* (“Freude”), *contempt* (“Verachtung”), *desperation* (“Verzweiflung”), and *sadness* (“Trauer”); one term was selected as relevant for suppressed facial expression: *seriousness* („Ernst“). In addition, participants rated the *intensity of expressivity* (“Ausdrucksintensität”). Originally, we intended to include evaluations on the item *ineffability/indeterminacy* (“Unbestimmtheit/Das Unbestimmbare”) in the analyses. The last term refers to the fact that composers deliberately express something that transcends the effable and therefore cannot sufficiently be translated into language.

**III) Table S2**

*Musical Excerpts*

| Stim. No. | Composer | Piece | Selection (Bars) | Opus | Singer No. |
|---|---|---|---|---|---|
| 1 | Jaques Offenbach | Song and scene „Es war einmal am Hofe von Eisenack“ | 163–173 | *Les Contes d' Hoffmann. Opéra fantastique en 4 actes. Piano reduction*, Paris 1907, p. 56. | 1 |
| 2 | Giacomo Puccini | Third act, Aria „Addio, fiorito asil” | 26–29 | *Madama Butterfly* SC 74, Score, Milan 1907, p. 440. | 1 |
| 3 | Giacomo Puccini | Atto Secondo. Third act, Aria „Addio, fiorito asil” | 22–24 | *Madama Butterfly* SC 74, Score, Milan 1907, p. 439. | 1 |
| 4 | Benjamin Britten | Song “Johnny” | 39–41 | *Cabaret Songs. For voice and piano*. London 1980, p. 14 | 2 |
| 5 | Benjamin Britten | Song “Johnny” | 27–37 | *Cabaret Songs. For voice and piano*. London 1980, p. 14 | 2 |
| 6 | Georg Friedrich Händel | Scene V, Aria “Scenes of horror” | 40–45 | *Jephta*, Leipzig 1886, Score, Ausgabe der deutschen Händelgesellschaft, p.72 | 2 |
| 7 | Georg Friedrich Händel | Scene V, Aria “Scenes of horror” | 70–79 | *Jephta*, Leipzig 1886, Score, Ausgabe der deutschen Händelgesellschaft, p.72 | 2 |
| 8 | Robert Schumann | “Ich grolle nicht” | 26–30 | *Dichterliebe* Op. 48, Heft 1, No. 7, Leipzig ca. 1844, p.15. | 3 |
| 9 | Richard Strauss | „Breit über mein Haupt“ | 8–12 | *No. 2 from 6 Lieder aus Lotosblätter* Op. 19, München 1897, pp.3–4. | 3 |
| 10 | Richard Strauss | „Breit über mein Haupt“ | 12–14 | *No. 2 from 6 Lieder aus Lotosblätter* Op. 19, München 1897, p.4. | 3 |

| | | | | | |
|---|---|---|---|---|---|
| 11 | Richard Strauss | „Breit über mein Haupt | 14–19 | *No. 2 from 6 Lieder aus Lotosblätter* Op. 19, München 1897, p.4. | 3 |
| 12 | Gustav Mahler | „Wer hat das Liedlein erdacht?“ | 58–67 | *No. 4 from Des Knaben Wunderhorn*, Score, Wien 1905, pp.69–70. | 4 |
| 13 | Gustav Mahler | „Wer hat das Liedlein erdacht?“ | 46–54 | *No. 4 from Des Knaben Wunderhorn“*, Score, Wien 1905 pp.68–69. | 4 |
| 14 | Wolfgang Amadé Mozart | Cavatine „Porgi, amor, qualche ristoro“ | 34–36 | *Le Nozze di Figaro,* Act II, No. 10, Kassel 1973, (NMA 5/2/16,1), p.164. | 5 |
| 15 | Robert Schumann | „Seit ich ihn gesehen“ | 18–23 | *N o. 1 from Frauenliebe und Leben*, Op. 42, Leipzig 1858, p.5 | 5 |

## IV) Table S3

*Most Relevant Emotional Expressions for each Stimulus (1–15) From a Pool of Ten Content Items*

| Evaluative items | Stimulus No. | | | | | | | | | | | | | | |
|---|---|---|---|---|---|---|---|---|---|---|---|---|---|---|---|
| | 1 | 2 | 3 | 4 | 5 | 6 | 7 | 8 | 9 | 10 | 11 | 12 | 13 | 14 | 15 |
| Anger | X | X | X | | X | X | X | | | X | X | X | X | X | X |
| Cheekiness | X | X | | | X | X | | X | X | X | X | X | X | X | |
| Disappointment | | X | X | X | | X | X | X | | | X | X | | | X |
| Tenderness | | X | X | X | | X | X | | X | X | X | | X | X | X |
| Pain | X | X | X | X | | X | X | X | X | X | X | X | | X | X |
| Longing | X | X | X | X | X | X | X | X | X | X | X | X | X | X | X |
| Joy | | | | | X | X | | | | | X | | X | | X |
| Contempt | X | | | | | X | | X | | | | X | | | |
| Desperation | X | X | X | | | X | X | X | X | X | X | X | | X | X |
| Sadness | X | X | X | X | | X | X | X | | X | X | X | | X | |

*Note*. All cells per column containing an “X” were included in the composite score, whereas empty cells were excluded. See Appendix Table S1 for a list of stimuli.

## V) Figure S1

Histograms for the ratings of crossmodal stimuli in the expressive face condition for laypersons and experts. The distribution of expressive intensity was right skewed, but the content-based emotion categories showed high numbers of “not-at-all” ratings. The evaluations of seriousness and expressive intensity were analyzed separately. The other evaluations contributed to the composite score of the emotion expression. Data include ratings of 15 stimuli from the 34 laypersons or 32 experts.

*(A) Laypersons*

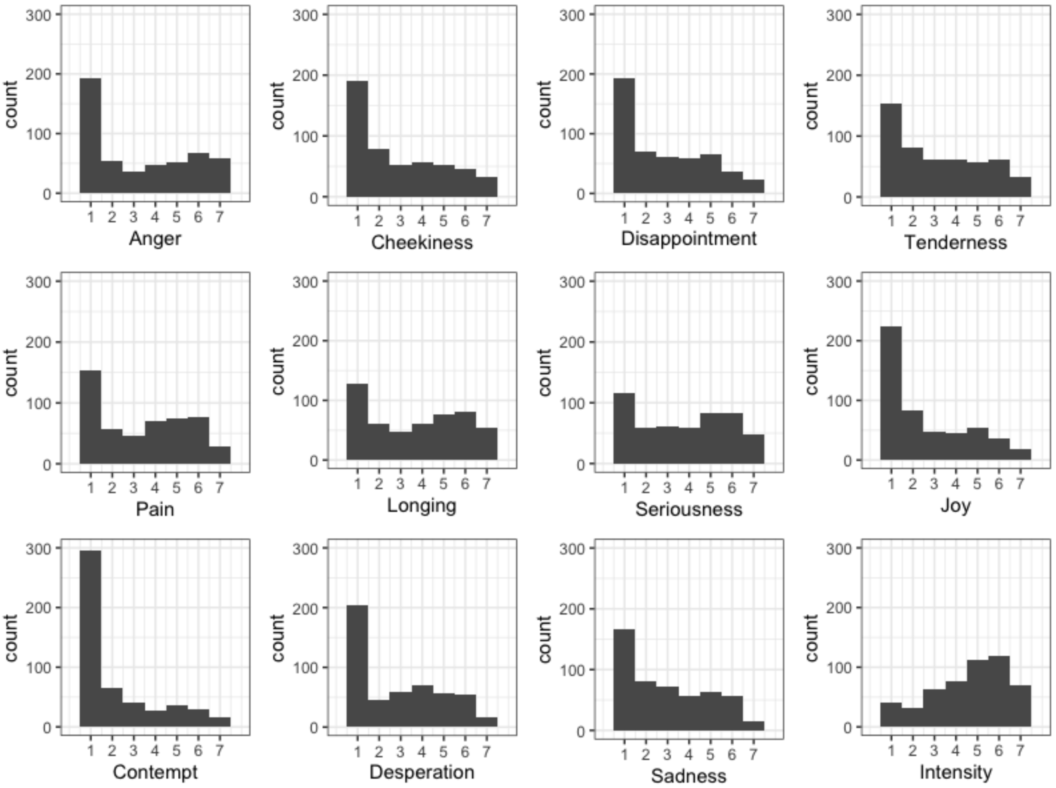

*(B) Experts*

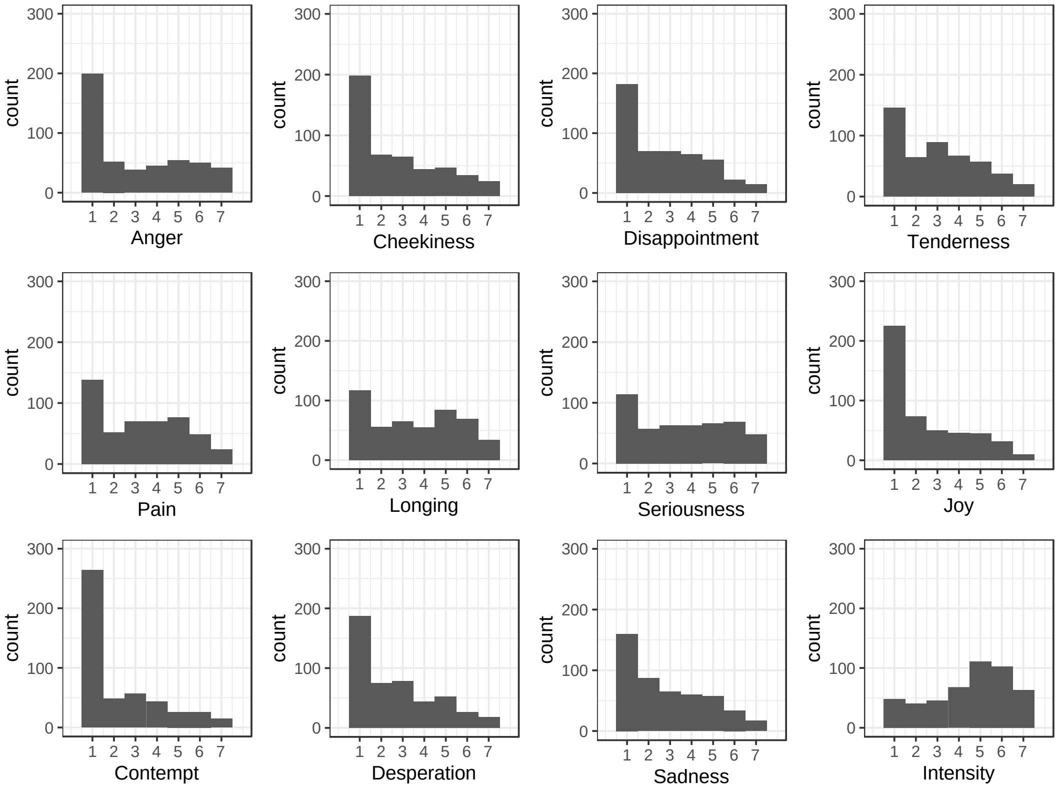


## VI) Figure S2

*Histograms for the Composite Scores of Emotion Expression (Crossmodal Stimuli, Expressive Faces)*

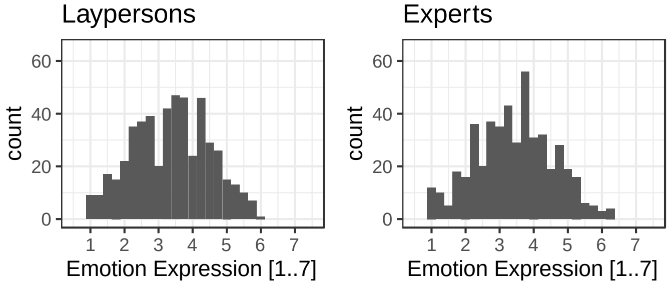


*Note*. Data include ratings of 15 stimuli from the 34 laypersons (left) or 32 experts (right) for crossmodal stimuli in the expressive face condition (A1V1).

## Results

### VII) Report of the Three-way ANOVAs Taking all Three Presentation Modes into Account at the Same Time

As a supplement, we provide here results that take all data depicted in Figure 3, main text, into account, fitted in a three-level ANOVA with the factors presentation mode (A, V, AV), facial expression (expressive, suppressed), and expertise.

**Intensity as Dependent Variable**

**Table S4**

*Results of the Two-Factor ANOVA including all Presentation Modes.*

| | *F* | *df*s | *p* | $\eta^2$ or $\eta_p^2$ |
|---|---|---|---|---|
| Presentation mode (A, V, AV) | 4.56 | 2, 128 | .012* | .066 |
| Presentation mode x Expertise | 2.94 | 2, 128 | .057 | 0.44 |
| Facial expression | 130.62 | 1, 64 | <.001* | .671 |
| Facial expression x Expertise | 8.65 | 1, 64 | .005* | .119 |
| Presentation mode x Facial expression | 32.02 | 2, 128 | <.001* | .333 |
| Presentation mode x Facial expression x Exp. | .87 | 2, 128 | .421 | .013 |
| Expertise | 1.24 | 1, 64 | .269 | .019 |

**Emotion Expression (Composite Score) as Dependent Variable**

**Table S5**

*Results of the Two-Factor ANOVA including all Presentation Modes.*

| | *F* | *df*s | *p* | $\eta^2$ or $\eta_p^2$ |
|---|---|---|---|---|
| Presentation mode (A, V, AV) | 21.04 | 2, 128 | <.001* | .247 |
| Presentation mode x Expertise | 1.08 | 2, 128 | .342 | .017 |
| Facial expression | 146.76 | 1, 64 | <.001* | .696 |
| Facial expression x Expertise | 8.99 | 1, 64 | .004* | .123 |
| Presentation mode x Facial expression | 24.59 | 2, 128 | <.001* | .278 |
| Presentation mode x Facial expression x Exp. | 2.64 | 2, 128 | .076 | .040 |
| Expertise | .215 | 1, 64 | .645 | .003 |

**Seriousness as Dependent Variable**

**Table S6**

*Results of the Two-Factor ANOVA including all Presentation Modes.*

| | *F* | *df*s | *p* | $\eta^2$ or $\eta_p^2$ |
|---|---|---|---|---|
| Presentation mode (A, V, AV) | 24.79 | 2, 128 | <.001* | .279 |
| Presentation mode x Expertise | 2.68 | 2, 128 | .072 | .040 |
| Facial expression | 7.45 | 1, 64 | .008* | .104 |
| Facial expression x Expertise | 0.60 | 1, 64 | .441 | .009 |
| Presentation mode x Facial expression | 16.63 | 2, 128 | <.001* | .206 |
| Presentation mode x Facial expression x Exp. | 1.21 | 2, 128 | .303 | .018 |
| Expertise | 0.01 | 1, 64 | .945 | .000 |

## VIII) Reliability of Evaluations

We provide here information on the reliability of evaluations (Shrout & Fleiss, 1979; ICC(2,1)). We used different ways to calculate ICCs simply to make our results comparable to other studies. However, we think that the first account is the most appropriately one. For the first account, we calculated ICCs to estimate inter-rater agreement, with *k* raters and 15 objects (stimuli) for each mode of presentation and each of two interpretations (expressive, suppressed facial expression) and each scale (eleven content scales, one intensity scale) separately. ICCs were based on individualized z-scores of the raw ratings. We decided on separating ratings due to the nested structure of the data (full repeated measures design). This account results in separate ICCs for each item of the scale for different conditions (presentation mode, facial expression). We also report the mean for the specific conditions across the eleven content-based items and the means for specific ratings across the different conditions (presentation mode, facial expression). Second, we calculated ICCs but did not take the nested structure into account. ICCs were calculated across all scales and stimuli, but separately for each condition of the full 3-by-2 (presentation mode; facial expression) design. All calculations of the ICCs were done in *R* (R Core Team, 2019) with the *irr* package (Gamer, Lemon, Fellows, & Singh, 2019) as two-way random effects models, and reliability was defined as inter-rater agreement.

**Table S7**

*Reliability Measure as Agreement between Participants across Stimuli (Laypersons)*

| | A0 | A1 | V0 | V1 | A0V0 | A1V1 | Mean [all modes] |
|---|---|---|---|---|---|---|---|
| R1 | 0.04 | 0.08 | 0.10 | 0.15 | 0.04 | 0.11 | 0.09 |
| R2 | 0.05 | 0.06 | n.s. | 0.09 | 0.04 | 0.05 | 0.05 |
| R3 | 0.09 | 0.12 | 0.06 | 0.13 | 0.04 | 0.11 | 0.09 |
| R4 | 0.17 | 0.10 | 0.07 | 0.10 | 0.10 | 0.13 | 0.11 |
| R5 | 0.12 | 0.16 | 0.03 | 0.16 | 0.06 | 0.15 | 0.11 |
| R6 | 0.14 | 0.16 | 0.04 | 0.06 | 0.09 | 0.07 | 0.09 |
| R7 | 0.09 | 0.08 | n.s. | 0.11 | n.s. | 0.15 | 0.07 |
| R8 | 0.08 | 0.08 | n.s. | 0.14 | n.s. | 0.07 | 0.06 |
| R9 | 0.09 | 0.06 | 0.14 | 0.19 | 0.04 | 0.15 | 0.11 |
| R10 | 0.10 | 0.12 | 0.04 | 0.18 | 0.03 | 0.13 | 0.10 |
| R11 | 0.10 | 0.03 | 0.05 | 0.24 | 0.08 | 0.16 | 0.11 |
| Mean [R1 to R11] | 0.10 | 0.10 | 0.05 | 0.14 | 0.05 | 0.12 | 0.10 |
| R12 | 0.04 | 0.09 | 0.05 | 0.07 | n.s. | 0.10 | 0.07 |

*Note.* ICCs (agreement) based on z-scores within participants for each of the evaluative items (eleven content item, one intensity item) across 15 stimuli and 34 laypersons; n.s.= no significant ICC that is the ICC is not different from zero, $p < .05$ (included as zero in row or column means). R1 to R11 denote the eleven content items: 1–anger, 2–cheekiness, 3–disappointment, 4–tenderness, 5–pain,

6–longing, 7–seriousness, 8–joy, 9–contempt, 10–desperation, 11–sadness; R12 was the intensity rating.

**Table S8**

*Reliability Measure as Agreement between Participants across Stimuli (Experts)*

| | A0 | A1 | V0 | V1 | A0V0 | A1V1 | Mean [all modes] |
|---|---|---|---|---|---|---|---|
| R1 | 0.07 | 0.14 | 0.15 | 0.18 | 0.08 | 0.18 | 0.13 |
| R2 | n.s. | 0.03 | n.s. | n.s. | n.s. | n.s. | 0.01 |
| R3 | 0.07 | 0.12 | 0.04 | 0.14 | 0.03 | 0.06 | 0.08 |
| R4 | 0.06 | 0.07 | 0.04 | 0.05 | n.s. | 0.06 | 0.05 |
| R5 | 0.18 | 0.15 | 0.03 | 0.15 | 0.12 | 0.15 | 0.13 |
| R6 | 0.09 | 0.11 | n.s. | 0.10 | 0.06 | 0.03 | 0.07 |
| R7 | 0.09 | 0.14 | 0.02 | 0.16 | 0.04 | 0.21 | 0.11 |
| R8 | 0.05 | 0.06 | 0.04 | 0.10 | n.s. | 0.14 | 0.07 |
| R9 | 0.12 | 0.14 | 0.08 | 0.14 | 0.03 | 0.10 | 0.10 |
| R10 | 0.13 | 0.22 | n.s. | 0.11 | 0.04 | 0.21 | 0.12 |
| R11 | 0.11 | 0.13 | 0.04 | 0.14 | 0.06 | 0.19 | 0.11 |
| Mean [R1 to R11] | 0.09 | 0.12 | 0.04 | 0.12 | 0.04 | 0.12 | 0.09 |
| R12 | 0.04 | 0.09 | 0.05 | 0.07 | n.s. | 0.10 | 0.07 |

*Note.* ICCs (agreement) based on z-scores within participants for each of the evaluative items (eleven content items, one intensity item) across 15 stimuli and 32 experts. This Table S5 is analogous to Table S4. Even not reported here, confidence intervals were rather large for the data of both groups, numerical difference between laypersons and experts are mostly within confidence ranges of the estimate. Some commonalities seem to show in both data set: Some variables seem to result in higher agreement (1–anger, 5–pain, 9–contempt, 10–desperation, 11–sadness) and other lower (2–cheekiness, 8–joy), in this respect, negative emotions seem to be easier to decode than positive emotions; reliability seems to be higher when expressive faces are presented (V1, A1V1) in comparison to when expressions are suppressed (V0, A0V0), but are about the same for visible expressive faces (V1, A1V1) and the auditory stimuli (A0, A1); content-based items (R1 to R11) seems to have higher overall reliability than the intensity rating (R12).

**Table S9**

*Reliability Measure as Agreement between Participants across Stimuli and Evaluations Using Individualized z-scores for Laypersons and Experts*

| | A0 | A1 | V0 | V1 | V0A0 | A1V1 |
|---|---|---|---|---|---|---|
| Laypersons | 0.16 [0.13-0.20] | 0.16 [0.13-0.19] | 0.14 [0.11-0.18] | 0.17 [0.14-0.21] | 0.15 [0.12-0.19] | 0.15 [0.12-0.19] |
| Experts | 0.14 [0.12-0.18] | 0.18 [0.15-0.22] | 0.15 [0.12-0.18] | 0.14 [0.11-0.18] | 0.12 [0.10-0.15] | 0.16 [0.13-0.20] |

*Note.* ICCs are reported with the confidence intervals in brackets. When comparing results to Table S4 and S5, the reliability measures in Table S6 are slightly higher than the mean (R1–R11) and more similar between conditions and groups.

**References**

Gamer, M., Lemon, J., Fellows, I., & Singh, P. (2019). *Irr: Various coefficients of interrater reliability and agreement*. R package Version 0.84.1. https://rdrr.io/cran/irr/

R Core Team (2019). *R: A language and environment for statistical computing*. Version 3.3.1. R Foundation for Statistical Computing, Vienna, Austria. URL https://www.R-project.org/

Shrout, P. E., & Fleiss, J. L. (1979). Intraclass correlations: Uses in assessing rater reliability. *Psychological Bulletin, 86*(2), 420–428. DOI: 10.1037/0033-2909.86.2.420
